# The 40-Hz auditory steady-state response enhanced by beta-band subharmonics

**DOI:** 10.3389/fnins.2023.1127040

**Published:** 2023-02-24

**Authors:** Shunsuke Sugiyama, Tomoya Taniguchi, Tomoaki Kinukawa, Nobuyuki Takeuchi, Kazutaka Ohi, Toshiki Shioiri, Makoto Nishihara, Koji Inui

**Affiliations:** ^1^Department of Psychiatry, Gifu University Graduate School of Medicine, Gifu, Japan; ^2^Department of Anesthesiology, Nagoya University Graduate School of Medicine, Nagoya, Japan; ^3^Neuropsychiatric Department, Aichi Medical University, Nagakute, Japan; ^4^Multidisciplinary Pain Center, Aichi Medical University, Nagakute, Japan; ^5^Department of Functioning and Disability, Institute for Developmental Research, Aichi Developmental Disability Center, Kasugai, Japan; ^6^Section of Brain Function Information, National Institute for Physiological Sciences, Okazaki, Japan

**Keywords:** auditory steady-state response, oscillation, harmonic, magnetoencephalography, schizophrenia

## Abstract

The 40-Hz auditory steady-state response (ASSR) has received special attention as an index of gamma oscillations owing to its association with various neuropsychiatric disorders including schizophrenia. When a periodic stimulus is presented, oscillatory responses are often elicited not only at the stimulus frequency, but also at its harmonic frequencies. However, little is known about the effect of 40-Hz subharmonic stimuli on the activity of the 40-Hz ASSR. In the present magnetoencephalography study, we focused on the nature of oscillation harmonics and examined oscillations in a wide frequency range using a time-frequency analysis during the 6.67-, 8-, 10-, 13.3-, 20-, and 40-Hz auditory stimuli in 23 healthy subjects. The results suggested that the 40-Hz ASSR represents activation of a specific circuit tuned to this frequency. Particularly, oscillations elicited by 13.3- and 20-Hz stimuli exhibited significant enhancement at 40 Hz without changing those at the stimulus frequency. In addition, it was found that there was a non-linear response to stimulation in the beta band. We also demonstrated that the inhibition of beta to low-gamma oscillations by the 40-Hz circuit contributed to the violation of the rule that harmonic oscillations gradually decrease at higher frequencies. These findings can advance our understanding of oscillatory abnormalities in patients with schizophrenia in the future.

## 1. Introduction

Steady-state responses (SSRs) are evoked oscillatory responses driven by a train of stimuli delivered at a sufficiently high rate. SSRs can be recorded non-invasively using electroencephalography (EEG) and magnetoencephalography (MEG) in different sensory modalities. Auditory steady-state responses (ASSRs) reach maximum amplitude at approximately 40 Hz (Galambos et al., 1981; Picton et al., 2003), which is within the gamma-band frequency range (30–80 Hz). ASSRs have been interpreted as reflections of endogenous oscillatory activity representing auditory objects (Santarelli et al., 1995; Santarelli and Conti, 1999; Ross et al., 2002, 2005a). Therefore, the 40-Hz ASSR is considered a sensitive electrophysiological index for investigating intrinsic gamma-band oscillations (Tada et al., 2020; Sugiyama et al., 2021). Gamma oscillations have been associated with several cognitive functions (Fries, 2009; Uhlhaas et al., 2011) and inhibitory GABAergic interneurons (Cardin et al., 2009; Sohal et al., 2009); therefore, it has attracted the interest of researchers. In addition, abnormalities in gamma oscillations have been shown in patients with neuropsychiatric disorders such as schizophrenia (Uhlhaas and Singer, 2010; Hirano and Uhlhaas, 2021) and Alzheimer’s disease (Mably and Colgin, 2018; Traikapi and Konstantinou, 2021). Particularly, many EEG and MEG studies using ASSR have revealed reduced power and phase synchronization in response to 40-Hz stimulation in patients with schizophrenia (Kwon et al., 1999; O’Donnell et al., 2013), and one meta-analysis concluded that deficits in the 40-Hz ASSR occur frequently in schizophrenia (Thuné et al., 2016).

In a frequency domain representation, when a periodic stimulus that induces SSR is presented, brain oscillatory responses are elicited not only at the stimulus frequency, but also may occur at its harmonic frequencies, which are integral multiples of the stimulus frequency (Regan, 1966; Zhou et al., 2016). For example, an auditory stimulus with a repetition rate of 90 Hz will generate ASSRs at 90 Hz (the fundamental), 180 Hz (the second harmonic), 270 Hz (the third harmonic), etc., which have peaks of amplitude and/or phase synchronization in a frequency domain representation. In general, these oscillatory responses decrease as the harmonic number increases (Okawa et al., 2017; Retter et al., 2020, 2021). Although oscillations at harmonic frequencies are an essential part of SSRs, they have not been systematically addressed in studies using the frequency-tagging approach (Retter et al., 2021). Moreover, a relatively large number of reports have studied the harmonics in the steady-state visual evoked responses (visual SSRs) (Vialatte et al., 2010; Rossion, 2014; Norcia et al., 2015; Rossion et al., 2020). However, few studies have investigated the harmonics of ASSRs in detail. Ross et al. (2000) investigated the effect of modulation frequency on the amplitude and phase of ASSRs at 30 different stimulation rates between 10 and 98 Hz and reported that the oscillatory responses of harmonics near 40 Hz were predominant over the fundamental ASSR at modulation frequencies between 10 and 20 Hz. Other studies have also confirmed that the amplitude of oscillation at 40 Hz is greater than that of the fundamental ASSR for the stimulation rate of 20 Hz (Pastor et al., 2002; Sugiyama et al., 2022). These results suggest that auditory stimuli at the subharmonic frequency of 40 Hz may specifically enhance the 40-Hz ASSR. However, despite the special attention given to the 40-Hz ASSR in previous studies, the extent to which the subharmonics of 40 Hz enhance the activity of the 40-Hz ASSR remains unknown. In addition, repetition rates below 10 Hz have received little attention in studies using ASSRs (Tlumak et al., 2011). Therefore, gaining a thorough understanding of the underlying mechanisms of the 40-Hz ASSR is crucial, as it has begun to demonstrate clinical effects. To address this, in the present study we recorded the MEG signals emitted under repetitive auditory stimuli at the one-sixth (6.67 Hz), one-fifth (8 Hz), one-fourth (10 Hz), one-third (13.3 Hz), and one-half (20 Hz) subharmonics of 40 Hz, as well as those emitted under the fundamental of 40 Hz. Time-frequency analysis was performed to investigate the modulation of neural oscillations over a wide frequency range from 4 to 100 Hz.

## 2. Materials and methods

### 2.1. Subjects

We enrolled 23 healthy volunteers (9 women and 14 men) aged 24–62 (mean, 38.78). The subjects were not using any medications at the time of testing and had no history of mental or neurological illnesses or substance misuse in the previous 2 years. The patients had a hearing threshold of <25 dB at 1,000 Hz, which was assessed using an audiometer (AA-71, Rion, Tokyo, Japan).

### 2.2. Auditory stimulation

Auditory stimuli were induced using repeats of a brief pure tone. The pure tone was 800 Hz in frequency, and the sound pressure level was 70 dB. Six pure tones were played in durations of 150, 125, 100, 75, 50, and 25 ms (rise/fall, 5 ms). Each tone was repeated until the time threshold of 1,000 ms was reached. Therefore, there were six frequency conditions: 6.67, 8, 10, 13.33, 20, and 40 Hz. The sound stimulus was presented binaurally *via* earpieces (E-A-RTONE 3A, Aero Company, Indianapolis, IN, USA), and the sound pressure was controlled with an audiometer (AA-71, RION, Tokyo, Japan).

### 2.3. MEG recordings

Magnetic signals were recorded using a 306-channel whole-head MEG system (Vector-view, Elekta Neuromag, Helsinki, Finland) composed of 102 identical triple sensor elements. Three separate measurements of magnetic fields were obtained from each sensor element’s two orthogonal planar gradiometers, one magnetometer, and multi-superconducting quantum interference device. The MEG signals were recorded using 204 planar-type gradiometers. Signals were captured using a 0.1–300 Hz band-pass filter and were digitized at 1,000 Hz. Epochs with MEG signals of >2.7 pT/cm were excluded from the average.

The experiments were conducted in a silent, magnetically protected room. The participants were instructed to sit in a chair and watch a silent movie projected on a screen 1.5 m in front of them and to ignore the auditory stimulation. The six auditory stimuli were randomly presented with an even probability using a stimulus onset asynchrony of 1,500 ms. A total of at least 100 artifact-free epochs were averaged for each stimulus per participant.

### 2.4. Data analysis

Signal space projection was used to suppress environmental noise. Artifacts due to eye blinks or the heart were removed using independent component analysis if visually detectable. Then, dipole analyses were performed to estimate an equivalent current dipole for the main component of the ASSR by hemisphere. In the present study, only dipole analysis was used for source estimation. For dipole analyses, the Brain Electrical Source Analysis software package (GmbH, Gräfelfing, Germany) was used. The MEG waveforms of the 40-Hz condition were averaged across 100 trials and were digitally filtered with a band-pass filter of 37.5–42.5 Hz. The equivalent current dipole for the main component of the ASSR was estimated per hemisphere in a time window of 200–1,000 ms. The left and right dipoles for the main components of the 40 Hz ASSR were estimated for all 23 subjects. However, in some cases, we could not estimate the dipoles in other conditions. Therefore, as in a previous study (Sugiyama et al., 2022), the obtained two-dipole model of the 40 Hz condition was applied to the MEG signals in all conditions, and time-frequency analysis was performed on the source strength waveforms obtained by the two-dipole model. The dipoles were estimated to be located in the transverse gyrus. By applying the two-dipole model to the MEG signals, source strength waveforms were obtained for time-frequency analysis. Time-frequency analysis was applied to each epoch at 250 ms before and 1,000 ms after the onset of auditory stimulation, and the amplitude and inter-trial phase coherence (ITPC) of evoked oscillations for each frequency were calculated from 4 to 100 Hz, with 1-Hz frequency resolution using Morlet wavelet transformation every 50 ms. Results of the analysis were then averaged throughout all epochs. For statistical analyses, the average amplitude and ITPC of the baseline from 250 to 0 ms before the onset of auditory stimulation (Pre) and the average amplitude and ITPC of 200–1,000 ms (Post) were used.

We performed three analyses. First, to confirm the trend that the amplitude and ITPC of oscillations decrease with increasing harmonic number, Post/Pre ratios from the first to fifth harmonics were compared without distinguishing the left and right hemispheres in five conditions (not the 40-Hz condition) using one-way repeated-measures analysis of variance (ANOVA) with harmonic as an independent variable. Here, the first harmonic was used as the fundamental stimulus frequency. The integer frequencies closest to the harmonic frequencies were used for the analysis under the 6.67- and 13.3-Hz conditions. Then, a two-way ANOVA was performed with hemisphere and PrePost as independent variables, to compare Pre and Post at 4–100 Hz under each condition to examine whether a distinct signal-to-noise ratio by harmonics was obtained for amplitude and ITPC, respectively. *Post hoc* paired comparisons were performed using Bonferroni adjusted *t*-tests when the hemisphere × PrePost interaction effects were significant. Post/Pre ratios of amplitude and ITPC at 40 Hz for conditions with significant increases were compared using two-way ANOVA with hemisphere and stimulus frequency as independent variables, respectively. To compare the differences between the conditions, *post hoc* multiple comparisons were performed using Bonferroni adjusted *t*-tests. All statistical analyses were performed at *p* ≤ 0.05.

## 3. Results

**FIGURE 1:**
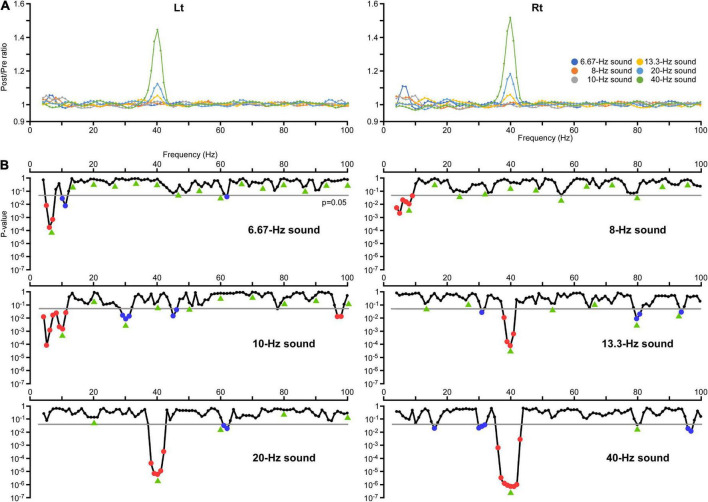
Changes in the amplitude of oscillations. **(A)** The effects of modulation of the oscillation on the amplitude at all frequency sampling points. The vertical axis indicates the ratio of the average amplitude at 200–1,000 ms (Post) relative to the baseline from 250 to 0 ms before the onset of auditory stimulation (Pre). **(B)** The results of two-way ANOVA. The vertical axis indicates the *p*-values for the effect of PrePost obtained in each condition. Significant increases in the amplitude are indicated by red dots and decreases are indicated by blue dots. Green arrows indicate harmonic frequencies in each condition.

**FIGURE 2:**
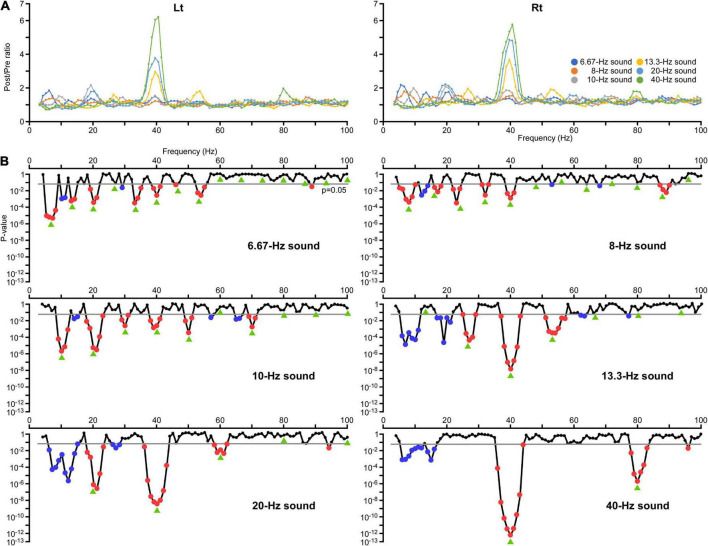
Changes in the inter-trial phase coherence (ITPC) of oscillations. **(A)** The effects of modulation of the oscillation in ITPC at all frequency sampling points. The vertical axis indicates the ratio of the average ITPC at 200–1,000 ms (Post) relative to the baseline (Pre). **(B)** The results of two-way ANOVA. The vertical axis indicates the *p*-values for the effect of PrePost obtained in each condition. Significant increases in the amplitude are indicated by red dots and decreases are indicated by blue dots. Green arrows indicate harmonic frequencies in each condition.

The train of six pure tones induced an increase in the amplitude and ITPC of oscillations at almost all stimulus frequencies and some of its harmonic frequencies. The ratio of the average amplitude and ITPC relative to the baseline (Post/Pre ratio) for each frequency in all conditions are shown in Figures 1, 2, respectively. Regarding the changes in amplitude and ITPC of oscillations associated with harmonic numbers, one-way ANOVA showed that the harmonic significantly affected the amplitude [*F*(4,1145) = 16.35, *p* = 4.97 × 10^–13^] and the ITPC [*F*(4,1145) = 21.55, *p* = 3.67 × 10^–17^]. Although the ITPC was smallest for the fifth harmonic, the first to third harmonics did not show a clear gradual decrease, which appeared to depend on the specific increase at 40 Hz (Figure 2A). Therefore, to confirm the phenomenon that oscillations decrease as the harmonic number increases, the data was reanalyzed without 40-Hz oscillation. As a result, we confirmed a gradual decrease in amplitude and ITPC of oscillations with advanced harmonics. One-way ANOVA showed that the harmonic significantly affected the amplitude [*F*(4,961) = 25.17, *p* = 7.50 × 10^–20^] and the ITPC [*F*(4,961) = 11.10, *p* = 8.42 × 10^–9^]. However, the data from each sound stimulus did not follow this trend.

**FIGURE 3:**
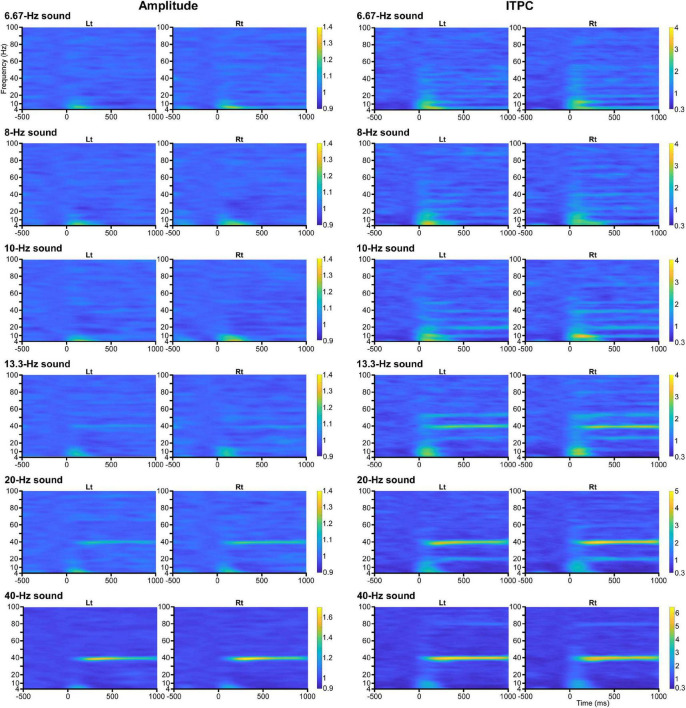
Time-frequency maps. Grand-averaged time-frequency maps of amplitude **(left panel)** and ITPC **(right panel)** to the baseline for each condition.

The effects of modulation of the oscillation in amplitude and ITPC at all frequency sampling points from 4 to 100 Hz were examined for each condition. Regarding the amplitude, two-way ANOVA (hemisphere × PrePost) revealed that PrePost significantly affected the oscillation amplitude around the first harmonic under the 6.67, 8, 10, and 40 Hz conditions. Under these four conditions, no significant increase in the amplitude of other harmonics was observed. In the 13.3 and 20 Hz conditions, a significant increase in the amplitude was observed around the third (38–41 Hz, *p* < 0.012) and second (38–42 Hz, *p* < 3.56 × 10^–4^) harmonics at 40 Hz, respectively, but not around the first harmonic (Figure 1B). As shown Figure 2A, the harmonics in each condition increased the ITPC in more frequency bands than the amplitude. The results of the two-way ANOVA showed that PrePost significantly affected the ITPC of oscillations around the harmonics: from the first to eighth harmonics in the 6.67-Hz condition, from the first to fifth harmonics in the 8-Hz condition, from the first to fifth harmonics and at the seventh harmonic in the 10-Hz condition, from the second to fourth harmonics in the 13.3-Hz condition, from the first to third harmonics in the 20-Hz condition, and at the first and second harmonics in the 40-Hz condition (Figure 2B). The time-frequency maps of amplitude and ITPC for each condition are shown in Figure 3. Notably, there was no significant increase in either the amplitude or ITPC around the first harmonic in the 13.3-Hz condition. Regarding the hemispheric difference, the overall amplitude tended to be greater for the left hemisphere under all conditions. In particular, a significant increase in amplitude was observed at 4–28 Hz under all conditions. This was largely due to the greater baseline amplitude in the left hemisphere. However, the degree of facilitation at the stimulus frequency tended to be greater in the right hemisphere. Conversely, the baseline ITPC tended to be greater for the right hemisphere. The facilitation at the stimulus frequency was also greater in the right hemisphere, which resulted in a significant difference around the stimulation frequency band; for example, at 36–42 Hz for the 40-Hz condition. Although no significant hemisphere × PrePost interactions were found in the amplitude, a significant interaction was observed around some harmonic frequencies in the ITPC; for example, at 39–42 Hz in the 20-Hz condition. The ITPC at 200–1,000 ms was significantly greater in the right hemisphere, but not at baseline.

A significant increase in ITPC was observed around 40 Hz for all conditions: in the 6.67 Hz (39–41 Hz, *p* < 0.023), 8 Hz (39–41 Hz, *p* < 0.004), 10 Hz (38–41 Hz, *p* < 0.013), 13.3 Hz (37–43 Hz, *p* < 0.027), 20 Hz (36–43 Hz, *p* < 0.019), and 40 Hz conditions (36–44 Hz, *p* < 0.048) (Figure 2B). We subsequently examined whether this increase in ITPC of the 40-Hz harmonic in each condition was significantly greater than the other harmonics. The Post/Pre ratios in the ITPC that were significantly increased for each condition were compared using two-way ANOVA with hemisphere and harmonic as independent variables. The results showed that the harmonic significantly affected the ITPC in the 6.67 Hz [*F*(6,17) = 6.46, *p* = 1.09 × 10^–4^], 13.3 Hz [*F*(2,21) = 9.30, *p* = 1.28 × 10^–4^], 20 Hz [*F*(2,21) = 24.13, *p* = 3.62 × 10^–6^], and 40 Hz conditions [*F*(2,21) = 24.13, *p* = 3.62 × 10^–6^]. Conversely, the harmonic did not play a role in determining the ITPC in the 8 and 10 Hz conditions (*p* > 0.067). The ITPC in the right hemisphere was significantly greater in the 20 Hz [*F*(1,22) = 9.86, *p* = 4.76 × 10^–4^] and 40 Hz conditions [*F*(1,22) = 5.43, *p* = 0.029]. There were no interaction effects in all conditions (*p* > 0.34). *Post hoc* multiple comparisons using Bonferroni adjusted *t*-tests revealed the following results: in the 6.67-Hz condition, the first harmonic was significantly higher than the fifth, seventh, and eighth (*p* < 0.030) harmonics, but the sixth harmonic (40 Hz) was not significantly different from the other harmonics (*p* > 0.16). In the 13.3-Hz condition, the third harmonic (40 Hz) was significantly increased compared to the second and fourth harmonics (*p* < 0.003). In the 20-Hz condition, the harmonics significantly increased in the order of the third, first, and second (40 Hz) (*p* < 0.001), and in the order of the second and first in the 40-Hz condition (*p* < 0.001).

There was a significant increase in amplitude around 40 Hz for the 13.3, 20, and 40 Hz conditions and for all conditions in the ITPC. The Post/Pre ratios of amplitude and ITPC at 40 Hz under these conditions were compared, respectively. In case of the amplitude, two-way ANOVA (hemisphere × stimulus frequency) revealed a significant difference in hemisphere [*F*(1,22) = 4.54, *p* = 0.044] and stimulus frequency [*F*(2,21) = 25.34, *p* = 2.52 × 10^–6^], but no interaction effect [*F*(2,21) = 3.47, *p* = 0.050]. All the amplitudes at 40 Hz in the three conditions showed right hemisphere dominance. *Post-hoc* tests showed that the amplitude at 40 Hz was significantly increased in the order of 13.3, 20, and 40 Hz conditions (*p* < 0.001). Regarding the ITPC, the results showed a significant difference in stimulus frequency [*F*(5,18) = 24.51, *p* = 1.90 × 10^–7^], but not between hemispheres [*F*(1,22) = 0.66, *p* = 0.43]. No interaction was detected between stimulus frequency and hemisphere [*F*(5,18) = 2.76, *p* = 0.051]. The *post-hoc* tests showed that the 40-Hz condition was the highest (*p* < 0.022), followed by the 20 and 13.3 Hz conditions. There was no significant difference between the 20 and 13.3 Hz conditions (*p* = 0.16), but they were significantly greater than the 6, 8, and 10 conditions (*p* < 0.001). There were no significant differences between the 6, 8, and 10 Hz conditions (*p* > 0.99).

However, reductions in amplitude or ITPC were detected in other frequency bands. For example, a significant decrease in the amplitude was observed; at 29–31 Hz in the 10-Hz condition (*p* < 0.016), at 31 Hz in the 13.3-Hz condition (*p* = 0.031), and at 30–32 Hz in the 40-Hz condition (*p* < 0.044) (Figure 1B). These reductions in amplitude were considered to reflect suppression of oscillations around 30 Hz by activating the 40-Hz oscillation, as shown in a previous study (Sugiyama et al., 2022). Additionally, a significant decrease in ITPC was widely observed around the alpha and beta frequency bands; at 10, 11, and 29 Hz in the 6.67-Hz condition (*p* < 0.017), at 12–14 Hz in the 8-Hz condition (*p* < 0.003), at 14 and 15 Hz in the 10-Hz condition (*p* < 0.025), at 6–11 and 17–21 Hz in the 13.3-Hz condition (*p* < 0.017), at 6–15 and 26–28 Hz in the 20-Hz condition (*p* < 0.044), and at 6–12 and 14–16 Hz in the 40-Hz condition (*p* < 0.014) (Figure 2B).

## 4. Discussion

In this study, with particular attention to harmonics, we examined brain oscillations over a wide frequency range during auditory stimulation between 6.67 and 40 Hz that have the harmonic at 40 Hz. Harmonics were identified in all conditions. In particular, the increase in ITPC by harmonics was detected at more frequencies than the increase in amplitude under all conditions. We first evaluated whether the oscillatory responses decreased as the harmonic number increased (Retter et al., 2021). However, the results did not follow this rule because the present study employed sound stimuli at subharmonics of 40 Hz. When the 40-Hz oscillation was excluded from the analysis, the response gradually decreased as the harmonic number increased, which was consistent with an exponential decrease (Retter et al., 2021). In each condition, both amplitude and ITPC of oscillations were significantly increased at each stimulus frequency for the 6.67, 8, and 10 Hz conditions, but only ITPC increased at stimulus frequency for the 20-Hz condition. However, neither amplitude nor ITPC of oscillations increased at the stimulus frequency for the 13.3-Hz condition. Interestingly, these two stimuli at 13.3 and 20 Hz caused a significant increase in the amplitude and ITPC of the 40-Hz oscillation relative to other harmonics, thus they specially enhanced the 40-Hz ASSR. These findings indicate that the brain oscillatory circuit in the auditory cortex is particularly driven by the 40-Hz sound stimulus, as well as by stimuli in the specific frequency band with a harmonic at 40 Hz, which is consistent with the previous study (Ross et al., 2000).

In the 6.67, 8, and 10 Hz conditions, the harmonic increases in ITPC were observed over a wide frequency range, but there were no harmonic increases in amplitude. The amplitudes at the stimulus frequency in these three conditions were significantly increased, but their Post/Pre ratios were very small compared to those in ITPC. It could be suggested that the harmonic increases in amplitude did not appear due to lower relative changes in amplitude at the stimulus frequencies. In a study examining the test-retest reliability of the ASSR using MEG, ITPC exhibited high reliability compared to spectral power (Tan et al., 2015). In addition, baseline-corrected spectral power for the ASSR at lower frequency (e.g., 5 Hz) resulted in lower reliability due to the higher baseline power at lower frequencies (Tan et al., 2015). Our results also suggest that ITPC has an advantage over amplitude in measuring harmonics and fundamentals in low-frequency oscillations. Conversely, in the 13.3-Hz condition, no significant increase at the stimulus frequency was observed not only in amplitude but also in ITPC. Even with the 20-Hz condition, there was no increase in amplitude at the stimulus frequency, only an increase in ITPC. These findings indicate that, at least between the 6.67 and 40 Hz conditions that were examined in this study, the 13.3-Hz oscillation circuit is the most difficult to drive, followed by the 20-Hz circuit. Picton et al. (1987) showed that responses for amplitude modulation tones were high at amplitudes between 2 and 7 Hz and between 27 and 55 Hz. Tlumak et al. (2011) reported the smallest fundamental amplitude at 10 Hz among auditory stimuli at 0.75, 1.25, 2.5, 5, 10, 20, and 40 Hz. These findings are roughly consistent with the present results.

In some conditions, the oscillatory responses did not follow the phenomenon of decreasing as the harmonic number increased, except the 40-Hz harmonic. In the 13.3-Hz condition, ITPC did not increase with stimulus frequency, but it did increase significantly not only in the third (40 Hz) but also in the second and fourth harmonics. This finding indicated that the special activation of the oscillation by harmonics affected not only 40 Hz but also the surrounding frequency bands. Conversely, ITPC under the 10-Hz condition showed that the second harmonic (20 Hz) was larger than the fundamental, although there was no significant difference between the fundamental and the harmonics. A similar phenomenon was observed in the 6.67-Hz condition, where the ITPC at the third (20 Hz) harmonic was slightly larger than the ITPC at the second harmonic. In addition, in the 8 and 10 Hz conditions, sporadic significant increases in ITPC were observed at the eleventh (88 Hz) and seventh (70th) harmonic, respectively. These trends may reflect a previous study that showed peaks in the phase response of the ASSR at approximately 20, 40, and 80 Hz (Ross et al., 2000). It has to be kept in mind, however, that the present results did not clearly show peaks around 20 and 80 Hz.

The 13.3 and 20 Hz conditions showed a specific increase in 40-Hz oscillations, but the amplitude of oscillations at the stimulus frequency did not show a significant increase. This indicates that these stimuli actually activated some oscillations at subharmonic frequencies of 40 Hz, but the response did not fall in a sufficiently narrow-frequency range to create a prominent peak. Thus, one explanation of the finding could be the non-linearity of the responsible cells (Retter et al., 2021). The lack of a significant increase in amplitude at 12–35 Hz in all conditions supports this view. Therefore, the non-linear frequency range appears to extend from the beta to low-gamma band. However, some results did not support this view, for example, ITPC of the 10-Hz stimulus was greater for the second harmonic (20 Hz) than that for the fundamental frequency. An alternative hypothesis to explain this phenomenon is that the activation of 40-Hz oscillation inhibits oscillatory activities, including 13.3 and 20 Hz. Our previous study demonstrated the suppression of ongoing low-gamma oscillations by the activation of 40-Hz oscillation (Sugiyama et al., 2022). In the present study, decreased oscillatory activity during the 40-Hz stimulus was observed at approximately 30–32 Hz. Numerous studies on patients with schizophrenia have demonstrated that broadband spontaneous oscillations increase during auditory stimulation at 40 Hz (Spencer, 2012; Hirano et al., 2015). An increase in 20-Hz oscillation for a 40-Hz drive has also been demonstrated in patients with schizophrenia (Spencer et al., 2008; Vierling-Claassen et al., 2008; Metzner and Steuber, 2021). These findings indicate that the 40-Hz stimulus can activate oscillations at broadband frequencies. Our hypothesis could explain why deficits in the 40-Hz oscillation in patients with schizophrenia are related to an increase in oscillations at broadband frequencies. Llinás et al. (1991) investigated guinea pig oscillatory neurons and demonstrated that neurons with intrinsic oscillatory properties were divided into broad-frequency oscillators (10–45 Hz) and narrow-frequency oscillators (35–50 Hz). The latter corresponds well to the 40-Hz oscillation in humans. Unlike the narrow-frequency oscillator, the broad-frequency oscillator often showed spontaneous oscillations, and its oscillatory frequency changed depending on the degree of membrane depolarization. Thus, it is possible, according to our hypothesis, that 40-Hz oscillation inhibits both spontaneous and evoked broadband oscillations. Interestingly, one *in vitro* study demonstrated the inhibition of low-gamma oscillations by a 40-Hz circuit (Middleton et al., 2008).

Our study has several limitations. First, whether the 40-Hz circuit suppresses broadband oscillations in patients with schizophrenia remains unexplored. Therefore, further clinical studies should be conducted to strengthen our hypothesis. Further research using cytological and other biological techniques is also needed to provide evidence that two categories of oscillators interact to generate neural oscillations. The second limitation concerns hemispheric differences. As shown previously (Ross et al., 2005b), the amplitude and ITPC of ASSR showed right hemisphere dominance. In contrast, while the overall baseline amplitude tended to be greater for the left hemisphere, the baseline ITPC tended to be greater for the right hemisphere. We could not find a reasonable explanation for the hemispheric difference between the amplitude and ITPC baselines. Furthermore, specifically designed studies are needed to address this issue. Finally, while significant reductions in ITPC were found in the alpha and beta frequency bands, the details of this finding are unclear. These reductions were common to all conditions, suggesting that they reflect the effect of the auditory stimulus rather than the stimulus frequency. They might also reflect event-related desynchronization by auditory stimuli (Krause, 2006; Pesonen et al., 2006).

## 5. Conclusion

Using the nature of oscillation harmonics, we identified subharmonics that specifically activate the 40-Hz ASSR. These subharmonics at the beta frequency band were not activated at their own stimulus frequency and could have been suppressed by the resonant 40-Hz circuit. These findings may provide novel insights into the pathophysiology of various neuropsychiatric disorders associated with abnormalities in the 40-Hz ASSR.

## Data availability statement

The original contributions presented in this study are included in the article/supplementary material, further inquiries can be directed to the corresponding author.

## Ethics statement

This study was approved by the Ethics Committee of the National Institute for Physiological Sciences, Okazaki, Japan and was conducted in accordance with the Declaration of Helsinki. The patients/participants provided their written informed consent to participate in this study.

## Author contributions

SS and KI designed the work and drafted the manuscript. SS, TT, TK, NT, MN, and KI performed the experiments. SS analyzed the data. KO and TS provided valuable critical input on the manuscript. All authors read and approved the manuscript.
